# Imaging analysis of Bartonella species in the skin using single‐photon and multi‐photon (second harmonic generation) laser scanning microscopy

**DOI:** 10.1002/ccr3.2939

**Published:** 2020-07-19

**Authors:** Azar Maluki, Edward Breitschwerdt, Lynne Bemis, Rosalie Greenberg, Bobak Robert Mozayeni, Jamie Dencklau, Marna Ericson

**Affiliations:** ^1^ Dermatology University of Minnesota Medical School ‐ Twin Cities Minneapolis Minnesota USA; ^2^ Dermatology College of Medicine University of Kufa Kufa Iraq; ^3^ Intracellular Pathogens Research Laboratory Center for Comparative Medicine and Translational Research College of Veterinary Medicine North Carolina State University(NCSU) Raleigh North Carolina USA; ^4^ Department of Biomedical Sciences University of Minnesota Medical School ‐ Duluth Campus Duluth Minnesota USA; ^5^ Medical Arts Psychotherapy Associates, PA. Summit New Jersey USA; ^6^ Founder and General Medical Director Translational Medicine Group PC North Bethesda Maryland USA

**Keywords:** Bartonella, confocal microscopy, psychiatric disorders, second harmonic generation, striae distensae

## Abstract

We demonstrate *Bartonella* spp are abundant in skin lesions resembling striae distensae. These striae distensae‐like lesions, coincidental with sudden onset of neuropsychiatric symptoms, indicate testing for suspected *Bartonella* spp. infection.

## Graphical abstract

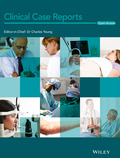

## 1 INTRODUCTION

*Bartonella* is a genus of Alphaproteobacteria within the family Bartonellaceae. They are re‐emergent, often‐neglected, stealth bacteria, with worldwide significance for zoonotic infections. *Bartonella* spp. are aerobic, gram‐negative, vector‐borne bacteria that can infect many different cell types including endothelial cells and erythrocytes of numerous mammalian hosts, causing prolonged infections in humans and animals with diverse disease manifestations.^1^, ^2^, ^3^ More than 38 spp. or subspecies have been described, 18 of which have been associated with an expanding spectrum of human diseases.^4^, ^5^, ^6^ *Bartonella quintana* has been detected in 4000‐year‐old human remains, representing the oldest evidence of this infection to human beings.^7^

*Bartonella* spp. are transmitted to humans by various blood‐sucking arthropod vectors and by animal bites and scratches.^8^, ^9^ Additional vectors, including sand flies, lice, fleas, biting flies and ticks, and potentially spiders ^5^, ^10^, ^11^ are known to transmit various *Bartonella* spp. to animals and humans. *Bartonella henselae* can multiply in the cat flea and persist in flea feces in the environment for at least 9 days.^8^, ^12^

After an intradermal inoculation by the vector, the *Bartonella* spp. infection may establish a primary dermal niche that likely includes the vascular endothelium. Inside the mammalian reservoir host, the infection spreads to the blood, a secondary niche, where bacteria invade erythrocytes and a long‐lasting bacteremia develops, a hallmark of *Bartonella* infection.^1^, ^13^, ^14^, ^15^, ^16^, ^17^ Bacteremia caused by *Bartonella* spp. is potentially fatal, especially in immunodeficient patients, while immunocompetent individuals are at risk of chronic infection.^18^ The establishment of chronic, stealth infection is achieved by evasion of innate immune responses. These include resistance to complement activation, antigenic variation of surface proteins, and inhibition of host cell apoptosis.^19^

There are no gold‐standard diagnostic tests to confirm *Bartonella* spp. infection. False‐negative results are frequent, even with multi‐step molecular and microbiological techniques and serology.^20^, ^21^ Thus, diagnosis of *Bartonella* spp. infections remains challenging, warranting development of sensitive and reproducible diagnostic methods.

In this study, we report the detection of *Bartonella*‐immunoreactivity in skin tissue biopsies of three subjects with psychiatric symptoms, using single‐photon confocal laser scanning microscopy; and the alteration in dermal collagen fiber organization in the nonclassical striae‐like skin track lesions using multi‐photon (SHG) laser scanning microscopy on multi‐stained thick tissue skin samples. Patients' blood and skin were also analyzed by PCR, hemi‐nested PCR, or enrichment culture.

## 2 CASE PRESENTATION

### 2.1 Patient – I

Seven‐year‐old male presented at a psychiatric clinic with mood swings, decreased need for sleep, oppositional behavior, and refusal to attend school. Subsequent diagnoses included bipolar disorder‐type II, obsessive‐compulsive disorder, separation anxiety disorder, sensory hypersensitivity, and seasonal affective disorder. He was treated with Omega‐3, mood stabilizers (Depakote), antianxiety medication (Buspar), and light therapy. At age 11, he developed severe headaches and stomach bloating. His school attendance remained erratic because of excessive fatigue. Evaluation for underlying infectious triggers revealed evidence of exposure to *B henselae*, *Borrelia burgdorferi*, and *Babesia microti*. At age 14, during antibiotic treatment for his tick‐borne infections, asymmetric striae‐like skin tracks appeared on his arms and back. *B henselae* and *B quintana* IFA titers were 1:256 against both strains, indicative of *Bartonella* exposure. Enrichment blood culture/PCR testing for *Bartonella* bacteria were negative in this patient.^22^ Using hemi‐nested PCR, *B henselae* DNA was amplified and sequenced from the skin tissue biopsy. Skin tissue biopsies were collected from a skin track lesion and from nonlesional skin and immunostained with anti‐Bartonella spp..^23^ Laser scanning microscopy indicated immunoreactive‐*Bartonella* spp.in both tissues (data not shown).

### 2.2 Patient – II

**FIGURE 1:**
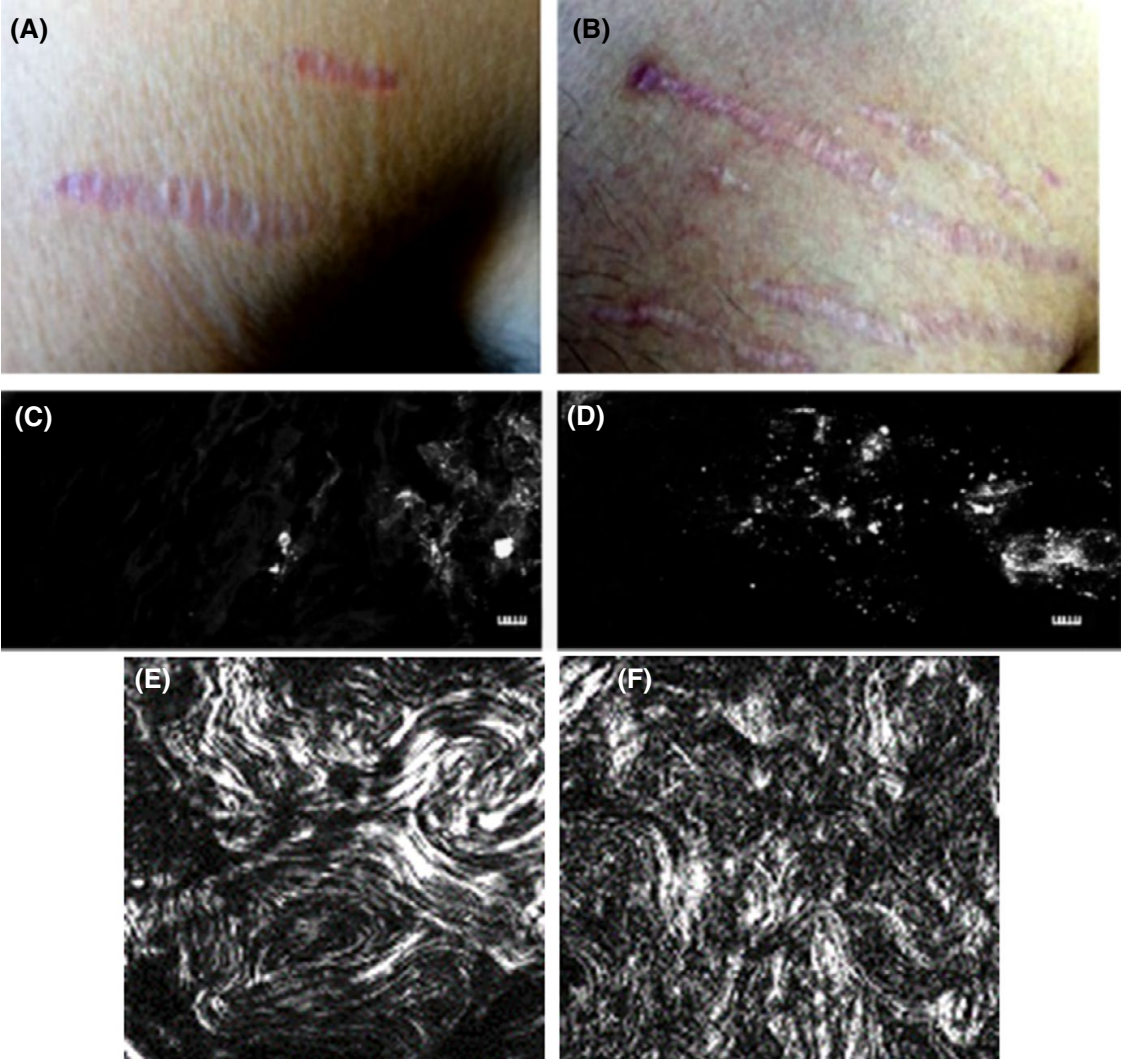
Striae‐like Bartonella skin tracks on the upper arms of patients with Bartonella spp. infection. Patient II. (Panels A, B) Axillary area from Patient II at two different time points, 6 mo apart from the distal side of the right shoulder depicts changes in color and patterning and unilateral distribution. Patients had no pain or itch associated with the lesions. This patient ingested no steroids, does not have EhlersDanlos, nor does he lift weights. (Panels C‐F) Skin punch biopsies were acquired from Patient II, one from adjacent nonlesional skin (Panels C, E) and the other from the leading edge of the lesion (Panels D, F). Biopsy sections were immunostained with antiBartonella henselae by imaged with single‐photon laser scanning microscopy (Panels C, D), scale bar = 5 microns). Qualitative image analysis reveals less immunoreactive B. henselae in nonlesional skin. (Panel C) vs, lesional (Panel D). All controls were negative—see supplemental information. Additionally, the stained sections were imaged using multi‐photon microscopy to visualize collagen fibrils in the dermis by second harmonic generation Panels E (nonlesional skin) and Panel F (lesional skin). Second harmonic generation (backward scatter) image data sets (single optical section) of collagen fibrils were more disordered and nonaligned, similar to scar tissue, compared to nonlesional collagen fibrils (Panel C). Scale bar = 50 microns, 25X objective

At age 9, this patient had depression which developed to major depressive disorder at age 14. As medications proved ineffective, he developed self‐injury behavior and increasing suicidal ideation with two psychiatric hospitalizations. His depression remained relatively unresponsive, despite a variety of psychiatric medication trials, multiple (Ketamine) infusions, several Transcranial Magnetic Stimulation sessions plus a variety of psychotherapeutic interventions. An integrative physician, who noted multiple striae‐like skin tracks on his torso and therefore suspected *Bartonella* as the causal agent, obtained *Bartonella* serology, and biopsies of a skin track lesion and nonlesional skin. Laser scanning microscopy revealed more immunoreactive‐*B henselae* in the lesional biopsy vs in the skin from the nonlesional biopsy. Second harmonic generation (SHG) microscopy revealed that the dermal collagen fibers of the lesional skin were scar‐like, disordered, and nonaligned (Figure 1). The *B henselae* IFA antibody titer was 1:256 and the *B quintana* IFA antibody titer was 1:128, indicative of *Bartonella* exposure. *Bartonella* DNA was not amplified by enrichment blood culture/PCR or hemi‐nested PCR from skin.

### 2.3 Patient – III

**FIGURE 2:**
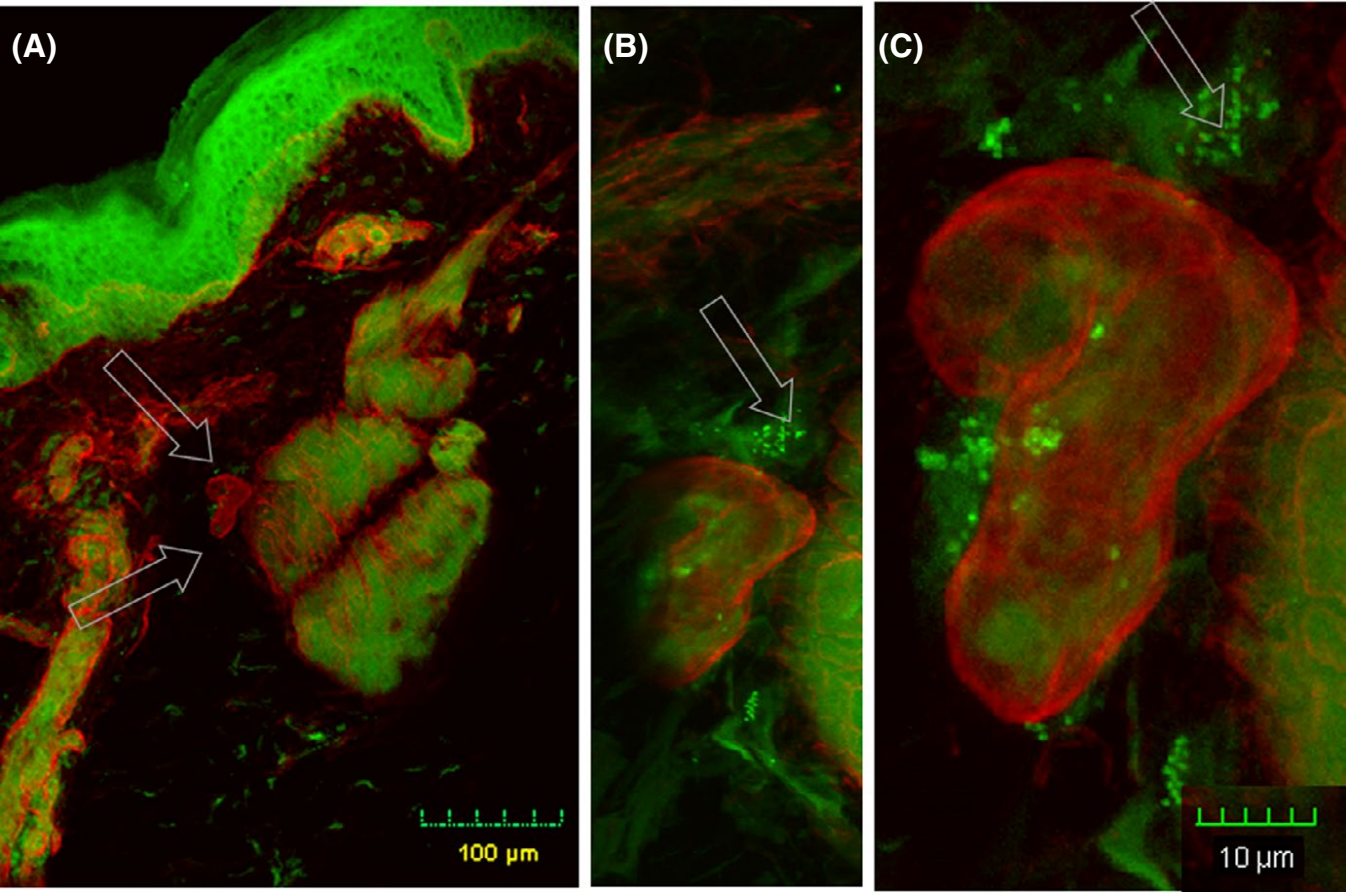
Immunoreactive‐B. henselae (green color, arrows) in the dermal layer of nonlesional skin from patient with persistent Bartonella spp. infection – Patient III. Eighteen‐year‐old male with persistent B. henselae, B. vinsonii subsp berkhoffii and Bartonella spp., infection shows most B. henselae‐ir outside of the vasculature (immuno‐positive collagen type IV – red color) in the dermis of nonlesional skin. A 4‐mm skin biopsy from the patient's calf area, a nonlesional area, was acquired. 100‐micron thick skin sections were stained using anticollagen IV (red) and anti‐Bartonella henselae (green). All controls were negative – see supplemental information. Z‐stack images collected using laser scanning confocal microscopy with 20X objective, 12 1.53‐micron optical sections (Panel A), 40X objective, 26 0.5‐micorn optical sections, (Panel B) and 60X objective with 2Z, 27 0.46‐micron optical sections {Panel C}

Fourteen‐year‐old male reported severe headache, sleeplessness, photophobia, short‐term memory loss, anxiety, and fatigue. He had a history of multiple tick bites and animal contact. Symptom severity prohibited school attendance. After a 2‐year duration illness and medical evaluations, the possibility of *Bartonella* spp. infection was considered. Blood samples were collected at different time points over the next 4 years. *Bartonella henselae* (San Antonio 2 strain type) and *B vinsonii* subsp *berkhoffii* (genotype I) DNA were PCR amplified and sequenced from his blood or enrichment blood cultures at multiple time points prior to and during the antimicrobial treatment period. Each blood specimen was processed using a multi‐PCR platform protocol with an insect cell culture‐based liquid growth medium for bacterial enrichment. Prior to beginning antibiotic therapy, a skin biopsy from a nonlesional area of the patient's calf was acquired, multi‐stained with *Bartonella* spp.‐specific antibody, and imaged using laser scanning confocal microscopy. *Bartonella henselae* immunoreactivity was visualized (Figure 2). After PCR results were obtained, different courses of antibiotics including varying combinations of doxycycline (oral and intravenous), rifampin, ciprofloxacin, azithromycin, atovaquone, rifabutin, clarithromycin, carbapenen, ceftriaxone, and silver were administered. Two and one‐half years later, he remained symptomatic and had an appendectomy. Though enrichment culture PCR results had been negative for 1.5 years, *Bartonella* spp. were again visualized in appendix tissue using the same laser scanning microscopy techniques. Four years after the symptom onset *Bartonella* spp. were visualized, immunoreactive *B henselae* was detected in a nonlesional skin fragment using the same methodology. Symptoms persist, but have decreased in severity, without additional antimicrobial interventions. Coinfection with *B* *henselae* and *B* *vinsonii* subsp *berkhoffii* was confirmed in the blood of this patient by PCR amplification and DNA sequencing. IFA titers were 1:256 to both strains. Patient reports he is functioning at 85% of normal.

## 3 DISCUSSION

In contrast to classical *striae distensae*, which are initiated by stretch, our data suggests that the Bartonella pathogen is disrupting the collagen fibrils in the dermis resulting in the observed Bartonella track formations. We propose the name Bartonella Tracks for these striae‐like lesions to distinguish them from classical *striae distensiae* and to emphasize causality and the association with psychiatric symptoms.

Previously, we reported using laser scanning microscopy to visualize immunoreactive‐*B henselae* and amplified‐*B henselae* DNA from a striae‐like skin track lesion of an 18‐year‐old son living with his family in the Netherlands. Both the son and his mother were infected with *B henselae* and both reported chronic symptoms for 3 years duration.^24^ The boy's skin was remarkable for large irregular tracks, similar to stretch marks, found on the legs, and the buttock. In that study, *B henselae* DNA was successfully amplified and sequenced from the boy's blood, serum, enrichment blood culture, and a skin track biopsy. Additionally, our research team reported the detection of *B henselae* immunoreactivity in human tissues from two patients with chronic *Bartonella* spp. infection, including brain tissue of an 11‐year‐old girl with cerebral vasculitis and infarction,^25^ and from the surgically excised femoral head of a 63‐year‐old female veterinarian with severe degenerative osteoarthritis involving the right hip joint.^26^ We were also able to visualize with multi‐photon laser scanning microscopy ^27^ in vitro invasion of mature erythrocytes by *B henselae* using real‐time imaging over 60 hours; a linear unmixing approach was used to separate the fluorescence emission spectra of human erythrocytes from native *B henselae* organisms.

In this study, we used multi‐photon laser scanning microscopy Second Harmonic Generation (SHG) imaging to determine the directionality and organization of the skin fibrillar collagen in the biopsies of both lesional and nonlesional skin. The 3D images created by SHG technology allow for delineation of collagen fiber arrangement in the dermis, while facilitating discrimination between normal and scarred skin tissue. SHG microscopy is ideal for the analysis and quantification of the spatial arrangement of collagen fibers in tissue and has emerged as a useful tool for studying key features of collagen remodeling.^28^ SHG imaging is an attractive alternative to conventional or fluorescent‐based histology techniques for visualizing the molecular structure of collagen due to its label‐free nature, high sensitivity and specificity. The optical sectioning capability of SHG also provides means of imaging tissue in 3D.

Clinical observations from physicians treating patients with chronic *Bartonella* spp. infection support evolving evidence that striae‐like skin track lesions can develop in different body areas during the course of their illness. *Bartonella‐*associated skin lesions are usually described as unilateral linear inflamed skin tracks that change in color and appearance with disease progression and in association with antimicrobial treatment. These skin lesions occur without preceding or accompanying predisposing factors, such as obesity, weight loss, growth spurts, excessive body tension, or corticosteroid intake. These clinical features of *Bartonella*‐associated skin tracks make them distinct from the traditional striae distensae, commonly known as stretch marks, which arise due to stretching of the dermis. Clinically, classical striae distensae involves the abdomen, breasts, buttocks and thighs, lumbosacral area and upper arms,^29^, ^30^, ^31^, ^32^ occurs frequently during times of rapid tissue expansion such as pregnancy, adolescence, obesity, bodybuilding, and in association with short or long‐term oral and topical corticosteroids.^31^, ^33^, ^34^ Additionally, striae distensae occur in a variety of other clinical settings including severe weight loss, cachexia and anorexia nervosa,^32^, ^35^ Cushing's and Marfan syndromes,^33^, ^35^ diabetes mellitus and in debilitating infections and illnesses such as chronic liver disease, tuberculosis and HIV infection.^31^, ^34^, ^36^ The patients in this study had none of these events.

There is increasing microbiologic evidence indicating bloodstream and cerebrospinal fluid infections with one or more *Bartonella* spp. in patients with neuropsychiatric symptoms.^36^, ^37^ In conjunction with the cases in this report, *B henselae* bacteremia may contribute to progressive, recalcitrant neuropsychiatric symptoms consistent with Pediatric Acute‐onset Neuropsychiatric Syndrome (PANS) in a subset of patients and may be misdiagnosed as schizophrenia.^37^ There is also evidence that *Bartonella* spp. infection may play a role in some cases of pediatric mood disorders.^19^ Consistent with these observations, all three patients in this study initially saw mental health professionals due to the psychiatric/psychological nature of the presenting symptoms. Mood disturbances, heightened anxiety, difficulty with school attendance and academic performance were the overwhelming clinical manifestations in each case. All three experienced a delay in treatment for their concomitant infection(s) for several months to a few years. The presence of the *Bartonella* spp. associated striae‐like skin tracks alerted clinicians to the possibility of *B* henselae in the differential diagnosis of these somewhat treatment resistant neuropsychiatric cases. This association requires more rigorous testing as it may be that mental health clinicians should consider asking about the presence of *Bartonella*‐associated skin tracks during their assessment of new patients or those that undergo changes in symptomatology over time.

It is also evident that currently no single diagnostic methodology will confirm *Bartonella* spp. infection in all patients. As an example, a recent study found that almost 50% of patients with nonspecific symptoms had positive *Bartonella* spp. serology and/or were *Bartonella* PCR/DNA sequence positive for one or more *Bartonella* spp. In that study, *Bartonella* spp. bacteremia was confirmed in one in four patients.^38^, ^41^ When *Bartonella* bacterial infection cannot be confirmed by PCR, diagnosis often relies on a combination of clinical, epidemiological, and serologic criteria.^25^, ^39^ In addition, it is possible that some bacteremic patients become anergic and do not produce a detectable IFA antibody response against the bacteria while antigenic variation among Bartonella strains could also result in false‐negative IFA results detected in some patients.^25^ Therefore, despite using multiple *Bartonella* antigens, serology can lack sensitivity, can only implicate prior *Bartonella* spp. exposure, may be associated with cross reactivity with members of other genera^40^ and these tests may be inaccurate in immunocompromised patients due to a diminished antibody response.^41^ Our ability to detect Bartonella DNA in the same tissue sample, using hemi‐nested PCR, proves an effective and valuable ancillary tool to detect and diagnose suspected *Bartonella* spp. infection. Laser scanning microscopy imaging of multi‐stained tissue biopsies has proven to be consistent, providing reliable detection of *Bartonella* spp and *Borrelia burgdorferi*. (data not shown). This technique has been primarily a research tool but we envision this technique in a diagnostic modality. This tool is currently commercially available for quantifying epidermal nerve fibers for diagnosing peripheral neuropathy. The work described here indicates the potential use of this imaging technique with a 2‐week turn‐around time. Indeed, it seems we need a toolbox of testing modalities to detect, diagnose and understand disease pathology in suspected *Bartonella* spp. infection.

## 4 CONCLUSION

In this case report, we used multiple techniques to detect *B henselae* infection in patients with striae distensae‐like lesions coincidental with sudden onset of neuropsychiatric symptoms. We used advanced imaging methods to characterize immunoreactive (ir)‐*B henselae in* skin biopsies, finding more ir‐*B henselae* in lesional vs nonlesional skin. Additionally, the imaging studies demonstrate dermal ir‐*B henselae* bacteria is found both within and outside of blood vessels. Second harmonic generation imaging (SHG) of dermal collagen fibrils, reveals that the dermal collagen in lesional tissue is disrupted and scar‐like. Advanced imaging of multi‐stained thick skin biopsies can help elucidate pathogenesis and may provide an important tool for diagnosis. Though this case report focused on describing the striae‐like lesions observed in patients infected with *Bartonella* spp. infections, we recognize the importance of coinfections as we and others expand our future studies to include other coinfections and diseases such as Morgellons disease.^42^ Importantly, the presence of striae‐like skin track lesions in patients presenting with neuropsychiatric symptoms indicate testing for suspected *Bartonella* spp. infection.

## CONFLICT OF INTEREST

Breitschwerdt EB in conjunction with Sushama Sontakke and North Carolina StateUniversity holds US Patent No. 7,115,385, Media and Methods for Cultivation of Microorganisms, which wasissued October 3, 2006. Breitschwerdt EB is a founder, shareholder and the chief scientific officer for GalaxyDiagnostics, a company that provides diagnostic testing for the detection of Bartonella spp. infection inanimals and human patients. The remaining authors have disclosed no conflicts of interest.

## AUTHOR CONTRIBUTIONS

AM, ME, and EB: designed the study. RG, EB, and RM: provided regents and clinical expertise. AM, JD, and ME: performed all microscopy related experiments. EB and LB: provided molecular analysis. All authors contributed equally to the writing process and accepted the final version.
